# *Helicobacter pylori* in oral cavity: current knowledge

**DOI:** 10.1007/s10238-024-01474-1

**Published:** 2024-09-04

**Authors:** Liana Cristina Melo Carneiro Costa, Maria das Graças Carvalho, Filipa F. Vale, Andreia T. Marques, Lucas Trevizani Rasmussen, Tsute Chen, Melina Barros-Pinheiro

**Affiliations:** 1 Programa de Pós-graduação em Ciências da Saúde, Campus Centro-Oeste Dona Lindu, Universidade Federal de São João del-Rei (UFSJ), Divinópolis, Brazil; 2 Departamento de Análises Clínicas e Toxicológicas da Faculdade de Farmácia da Universidade Federal de Minas Gerais (UFMG), Belo Horizonte, Brazil; 3 BioISI – BioSystems and Integrative Sciences Institute, Faculty of Sciences, Universidade de Lisboa, Lisbon, Portugal; 4 Faculty of Medicine of Marília (FAMEMA), São Paulo, Brazil; 5 The Forsyth Institute (Microbiology), Cambridge, MA USA; 6 Department of Oral Medicine, Infection and Immunity, Harvard School of Dental Medicine, Boston, MA USA; 7 Research Institute for Medicines (iMed.ULisboa), Faculty of Pharmacy, Universidade de Lisboa, Lisbon, Portugal

**Keywords:** *Helicobacter pylori*, Mouth, Oral cavity, Oral microbiota, Biofilm, Oral microbiology

## Abstract

The oral cavity may play a role as a reservoir and in the transmission and colonization of *Helicobacter pylori.* The route of transmission for *H. pylori* is not fully understood. The prevalence of this pathogen varies globally, affecting half of the world’s population, predominantly in developing countries. Here, we review the prevalence of *H. pylori* in the oral cavity, the characteristics that facilitate its colonization and dynamics in the oral microbiome, the heterogeneity and diversity of virulence of among strains, and noninvasive techniques for *H. pylori* detection in oral samples. The prevalence of *H. pylori* in the oral cavity varies greatly, being influenced by the characteristics of the population, regions where samples are collected in the oral cavity, and variations in detection methods. Although there is no direct association between the presence of *H. pylori* in oral samples and stomach infection, positive cases for gastric *H. pylori* frequently exhibit a higher prevalence of the bacterium in the oral cavity, suggesting that the stomach may not be the sole reservoir of *H. pylori*. In the oral cavity, *H. pylori* can cause microbiome imbalance and remodeling of the oral ecosystem. Detection of *H. pylori* in the oral cavity by a noninvasive method may provide a more accessible diagnostic tool as well as help prevent transmission and gastric re-colonization. Further research into this bacterium in the oral cavity will offer insights into the treatment of *H. pylori* infection, potentially developing new clinical approaches.

## Introduction

*Helicobacter pylori* is the first bacterium formally recognized as a bacterial carcinogen [1] and a pathogen transmitted independently of an individual’s symptoms [2]. *H. pylori* can be transmitted through different routes and patterns. These include fecal–oral, oral–oral, gastric–oral, anal–oral, and genital–oral routes, and person-to-person, animal-to-human, foodborne infection, and occupational exposure patterns. The most common routes of transmission are fecal–oral and oral–oral, and the most common pattern is person-to-person transmission [3]. According to the World Gastroenterology Organization (2021), *H. pylori* infection affects half of the world’s population. Its prevalence varies according to geographic region, ethnicity, race, age, and socioeconomic factors, being higher in developing countries. There is wide variation in the prevalence of infection between and within countries, within a single city, and even between subgroups within a population [4]. The oral cavity contains an estimation of over 750 bacterial species, many of which can cause local and systemic diseases [5, 6]. Swallowing can transfer oral bacteria into the stomach and influence the composition of the gastric. It was reported that the bacterial communities in the gastric fluid are closely related to those in the oral cavity [7, 8]. It is not clear how *H. pylori* functions in the oral microflora, but its presence in the oral cavity may cause an imbalance in the microbiome. Oral transmission of microorganisms to the stomach can shape or remodel the microbial ecosystem in both habitats. *H. pylori* infection was reported to have a major impact on the microbiome of the oral-intestinal axis [9].

The adaptation of *H. pylori* to different physiological habitats in the host may be responsible for the differences in its growth and pathogenicity [10]. The mechanism of *H. pylori* colonization in the oral cavity is not well understood. Detecting it with high sensitivity and specificity is difficult due to the presence of many bacterial species in the mouth [11]. The oral and nasopharyngeal cavities are potential reservoirs of *H. pylori*. Saliva, dental plaque, tongue, root canals, oral mucosa, and tonsillar tissues are extra-gastric reservoirs [12]. A study compared the presence of the bacterium in saliva between laryngopharyngeal reflux (LPR) and non-LPR groups, and the saliva demonstrated a higher presence of *H. pylori* in the LPR group [13]. In cases where gastric biopsies test negative for *H. pylori*, but oral samples test positive, it supports the idea that the oral cavity may serve as a natural reservoir [14]. There is no consensus between the presence of bacteria in the mouth and its relationship with infection of the gastric mucosa among scientific community. There is, however, a growing interest in exploring the oral cavity as a potential nesting site for *H. pylori*, potentially influencing the transmission process of this bacteria [15]. The presence of *H. pylori* and its virulence factors in oral cavity should receive more attention in research [16]. The diversity of *H. pylori* genotypes between stomach, feces, and saliva in the same patient suggests that more than one strain may exist in the saliva and stomach of the same patient due to co-infection or genetic variation [15, 17]. In addition to host characteristics, differences in virulence and genetic diversity may contribute to variable outcomes in *H. pylori* infection [18]. This article aims to provide a brief review of the prevalence of *H. pylori* in the oral cavity, the characteristics of the oral cavity that favor colonization, the virulence heterogeneity between strains, and noninvasive methods for detecting *H. pylori* in the mouth.

## Methodology

To fulfill our objective, a keyword-based survey was performed in PubMed, Cochrane, Google Scholar, Virtual Health Library (VHL), and ScienceDirect databases. We used the following descriptors in the research: “*Helicobacter pylori*,” “Diagnostic Techniques and Procedures,” “Oral *Helicobacter pylori*,” “mouth,” “oral cavity,” “Rapid Immunochromatographic Tests,” “Polymerase Chain Reaction,” “PCR,” “Urea Breath Test,” “Serological Test,” “Next Generation Sequencing,” and “Multi-locus Sequence Typing.” Only articles in English were selected. In the first stage, title and abstract of articles resulted from keyword search were reviewed, and full content of articles with high interest were further reviewed.

## Prevalence of *H. pylori* in the oral cavity

The prevalence of *H. pylori* infection is highest in adults in Africa, Eastern Mediterranean regions, Russia, Middle America, and South America. In children, the prevalence is lower than in adults in Russia, Western Pacific, and European regions. However, the prevalence of *H. pylori* infection is similarly high in children and adults in Africa, Eastern Mediterranean regions, and Middle America and South America [19]. The prevalence of *H. pylori* in the oral cavity can vary widely from 0 to 100%. This variation can be attributed to several factors, such as the characteristics of the studied population, sample collection methods, and the methodologies used to detect the microorganism [20, 21]. The prevalence of *H. pylori* in the oral cavity is significantly higher in patients positive for gastric *H. pylori* than in patients negative. It is also greater in the oral cavity in patients with clinical and/or histological gastroesophageal disease compared to patients with non-ulcer dyspepsia or healthy controls [22]. The abundance presence of *H. pylori* in supragingival plaque was observed among individuals with gastric dyspepsia and periodontal disease, as well as among individuals without gastric dyspepsia but with periodontal disease, when compared to those with gastric disease but without periodontal disease and individuals without gastric dyspepsia and no periodontal disease [23].

A comparison of samples collected from various locations within the oral cavity revealed variations in the prevalence of *H. pylori* among these locations [11, 24–27] and the results indicated that at least one type of oral sample was positive for *H. pylori* [24]. Comparing the detection rate and quantity of *H. pylori* in dental biofilm and saliva samples among studies is challenging due to various factors. These include differences in detection methods, studied population, patient age groups, oral health status, PCR primers used with varying sensitivity and specificity, clinical sample types and numbers, in addition to laboratory procedures [28]. The frequency of *H. pylori* presence varies depending on its location in the oral cavity, such as dental plaque, saliva, tongue, and dental pulp (Table 1). Studies addressing the prevalence of *H. pylori* in the oral cavity vary greatly in their design, and only some of them investigate both the prevalence in the oral cavity and in the gastric mucosa. Moreover, these studies utilize diverse diagnostic methods to ascertain gastric *H. pylori* infection status, and the accuracy of these methods varies, which limits inter-study comparisons. Additionally, although some studies detect *H. pylori* in both the oral cavity and the stomach, they do not always present the prevalence in the oral cavity according to the presence or absence of gastric infection, adding a level of difficulty to comparing these studies (Table 1). Despite these limitations, in most cases, the prevalence of *H. pylori* appears to be higher in individuals with gastric colonization by the bacterium (Table 1). This underscores the significance of studying *H. pylori* in the oral cavity for two main reasons. First, there is the potential for reinfection and the oral cavity can act as a reservoir for *H. pylori*, leading to reinfection of the stomach even after eradication therapy. Secondly, there is the impact on the oral microbiome. Colonization of the oral cavity by *H. pylori* can cause significant changes in the oral microbiome, which can have broader implications for oral and systemic health. Regarding the relationship between *H. pylori* in the oral cavity and its presence in the stomach, there are conflicting findings. Of the studies reviewed, some studies (33, 49, 93) did not find a statistically significant association between the presence of *H. pylori* in the stomach and the oral cavity, while others (17, 27, 79, 80, 88) demonstrated such an association. The data reviewed are controversial, and there is no general consensus. These results should be interpreted with caution, and further studies are needed.

**Table 1:** Positive detection of *Helicobacter pylori* in the oral cavity

References	Country or region	Number of samples	Oral Sample	Prevalence in oral sample (%)	Prevalence in oral cavity according to *H. pylori* infection in the stomach (%)		Gastric *H. pylori* infection detection method*
					*H. pylori* positive	*H. pylori* negative	
Song et al. [27]	Germany	117	Dental plaque	68.0	64.0	52.0	13C-urea breath test
		42	Saliva	55.0			
Chitsazi et al. [93]	Iran	88	Dental plaque	34.1	36.4	31.8	Gastric biopsy/RUT t
Mishra et at. [87]	India	245	Saliva	45.7	nd (a)	nd (a)	Stool/PCR (HSP60)
Eskandari et al. [88]	Iran	67	Dental plaque	5.9	17.4	0	Gastric biopsy/RUT
Silva et al. [92]	Brazil	30	Dental plaque	36.6	36.6	nd (b)	Gastric biopsy/PCR (16S rRNA), RUT and histology
			Saliva	53.3	53.3	nd (b)	
Silva et al. [79]	Brazil	30	Dental Plaque	20.0	nd (a)	nd (a)	Gastric biopsy/PCR (16S rRNA, *ureA*)
			Saliva	30.0			
Fernandez-Tilapa et al. [33]	Mexico	200	Saliva, Dental Plaque	17.0	18.5	14.5	Serology (Anti-*H. pylori* antibodies)
Momtaz et al. [17]	Iran	300	Dental Plaque	0	nd (a)	nd (a)	Gastric biopsy/RUT
			Saliva	10.7			
Ramón-Ramón et al. [29]	Mexico	196	Saliva	30.6	24.0	6.6	Gastric biopsy/PCR (16S rRNA), and histology
Amiri et al. [65]	Iran	45	Dental Plaque	77.8	–	–	nd
Ogaya et al. [82]	Japan	40	Saliva	0	-	-	nd
			Root canal specimens	15.0	–	–	
Ismail et al. [80]	UK	49	Dental Plaque	40.8	83.3	16.1	Gastric biopsy/histology
Tirapattanun et al. [62]	Thailand	118	Saliva	50.0–57.0	–	–	nd
Aksit Bıcaket al. [28]	Turkey	100	Saliva	40.0–94.8	75.9–94.8	75.0–83.3	Gastric biopsy/histology
			Dental Plaque	16.7–100	76.6–91.4	66.7–83.3	
Medina et al. [89]	Chile	61	Saliva, Dental Plaque	50. 8	50.8	nd (a)	Gastric biopsy/PCR (*ureA*)
Abu-lubad et al. 2017 [90]	Jordanian	60	Dental Plaque	100	–	–	nd
Nomura et al. [43]	Japan	131	Inflamed pulp specimens	3.1–38.9	–	–	nd
Wongphutorn et al. [31]	Thailand	110	Saliva	63.6	42.7	nd (a)	Stool/PCR (16S rRNA) and IFA
Iwai et al. [25]	Japan	192	Saliva, Dental Plaque, Dental Pulp	0; 1.0; 12.0	84.0	1.2	Urine (Anti-*H. pylori* antibody)
Kadota et al. [11]	Japan	39	Dental Pulp, Saliva, Extracted teeth	2.6; 5.1; 7.9	–	–	nd
Nagata et al. [26]	Japan	88	Tongue Coat, Saliva, Dental Plaque	2.3; 4.5; 36.4	–	–	nd
Mallikaarachchi et al. [81]	Sri Lanka	71	Dental plaque	9.9–28.95	–	–	nd
Jara et al. [49]	Chile	41	Oral mucosa	29.2	100	0	Gastric biopsy/RUT
		38	Saliva	36.8	27.3	33.3	
Mehdipour et. al. [91]	Iran	72	Dental plaque	20.8	–	–	nd
Chi et al. [24]	China	242	Saliva	80.2	nd (a)	nd (a)	Gastric biopsy/histology and culture
			Mouthwash	69.8			
			Dental Plaque	52.9			
Wongsuwanlert et al. [16]	Thailand	41	Saliva	85.4	84.8	nd (a)	Gastric biopsy/culture
			Dental plaque supragingival	86.7			
			Dental plaque sub-gingival	83.9			
Moosavian et al. [71]	Iran	106	Dental plaque	17.9	nd (a)	nd (a)	Gastric biopsy/RUT

*RUT rapid urease test, *IFA* Indirect immunofluorescence assay, *nd* not determined; (a) the prevalence of infection by *H. pylori* infection in stomach was determined, but the prevalence of *H. pylori* in oral cavity for infected and/or not infected patients was not discriminated; (b) all individuals were infected with *H. pylori*

## *H. pylori* heterogenicity and virulence

There is significant genetic diversity among strains of *H. pylori* that infect humans, and this diversity contributes to their virulence through genes like *cagA* and *vacA*. [15]. The *vacA* gene encoding a vacuolating cytotoxin is present in all *H. pylori* genomes and has two main variable parts, the signal or s-region, and the middle or m-region. The s-region is classified into s1 and s2 types and the m-region into m1 and m2 types [17, 30]. Differences in the s1 and s2 regions and the m1 and m2 regions lead to variations in the genotype and vacuolating activity of different *H. pylori* strains [17, 18]. Genotypes of the *vacA* gene often differ between oral cavity and intestinal tract samples. Investigation of the phylogeny of *H. pylori* in saliva and fecal samples based on DNA sequences within the conserved region of the *vacA* gene showed that oral and fecal strains belonged to different clusters in the phylogenetic tree. This suggests that certain strains of *H. pylori* may have preferred tissue sites for colonization, differential ability to survive within the gastrointestinal tract [31], or an adaptive response to local environment conditions. In another study, saliva, supra-, and sub-gingival plaque samples were examined for the presence of *cagA* and *vacA* genes. Of the strains tested, 52.6% were found to be positive for the *cagA* gene. It is more prevalent in gastric cancer patients than in non-cancer gastritis ones. When the adhesion ability was considered among the strains, the *cagA*-positive strains had significantly higher adhesion ability than the *cagA*-negative strains in all tested cell lines [16].

The frequencies of s1a allele are 58.3% and 52.7% in saliva and gastric samples, respectively. This allele sequence is the most frequent signal in this region. In saliva samples, 16.6% contain the s2 allele, whereas no gastric biopsy contains this allele. Saliva (83.3%) and gastric biopsy samples (50%) carry the m2 allele which is the most frequent middle region [32]. Disagreement was reported among gastric biopsy, saliva, and dental plaque regarding the presence of *cagA* and *vacA* genes and the association of their alleles with *H. pylori*. Only some patients exhibited the same genetic profile based on the analyzed genes, indicating a wide variety of strains and mixed colonization in the same host. Of the samples that tested positive for *cagA,* some were associated with *vacA* s1, some with *vacA* s2, and some with both s1 and s2. The middle region (m1 or m2) of the *vacA* gene was only genotyped in *H. pylori* gastric isolates which is likely due to the heterogeneity in the *vacA* gene. Patients with dental plaque-positive strains had the *cagA* gene and were associated with the toxin-producing *vacA* s1, while patients with *cagA*-negative samples demonstrated the *vacA* genotypes s1 [14]. The *vacA* alleles were typed in the samples from subjects without dyspepsia symptoms. The s1 allele was detected in 66.7% of oral samples, and vacA m1 and m2 alleles were found in 16.7% of oral samples [33].

The *vacA* genotype is commonly found in saliva and biopsy of the same patient, and a 51.1% of the saliva-positive/biopsy-positive patients presented the same genotypes in both sites [29]. A study compared *H. pylori cagA* and the *vacA* allelic status between saliva and gastric specimens in the same patients with dyspepsia. The *cagA* gene was found in 94% gastric biopsies and in 83% saliva samples [32]. It is important to determine whether oral and gastric *H. pylori* are genetically close to each other for future studies aiming to resolve the role of *H. pylori*. If the genetic similarity between the two is established, it would suggest that the infection could be triggered by either the mouth and the stomach simultaneously or the oral cavity could act as a reservoir for the bacteria. If *H. pylori* exists in the oral cavity and serves as a source of infection for the stomach, then eradication therapy for *H. pylori* should be carried out simultaneously in both the oral cavity and the stomach [34]. Results involving 10 virulence genes of *H. pylori* in saliva, mouthwash, and dental plaque, as well as in gastric mucosa, showed high consistency (> 78%) of *H. pylori* genotypes in saliva and gastric mucosa [24].

More studies are needed that not only confirm the presence of *H. pylori* but also determine the genetic makeup of *H. pylori* in both the oral cavity and the stomach. This is essential to establish the genomic similarity between oral and gastric strains of H. pylori. Whereas the strains that colonize the oral cavity and stomach could be the same, the elimination of oral *H. pylori* could prevent recurrent gastric infection.” Otherwise, if *H. pylori* in gastric and oral samples are different, it would suggest that the mouth is not a transient environment for *H. pylori* to pass through stomach but harbors its own distinct strains.

## *H. pylori* colonization and oral microbiome dynamics

It is likely that various strains of *H. pylori* reach the oral cavity through different routes, such as personal contact, vomiting, or gastroesophageal reflux, and that these strains remain in saliva and dental plaque long enough to reach the stomach. Therefore, oral colonization may serve as a reservoir of *H. pylori* and a potential source of stomach infection or reinfection [29]. The potential reasons for persistent *H. pylori* colonization include directional motility guided by chemoreceptors, acid niches neutralized by urease, regulation of host immunity, oral colonization, adaptive expression of mucins and adhesins, and the acid responsiveness of adhesion [35].

*H. pylori* is one of the HOMD taxa (taxon ID HMT-812). According to HOMD’s “Ecology” page based on the data from an oligotyping analysis of the human oral microbiome [74], *H. pylori* was found in 8 of the 9 oral sites analyzed, including buccal mucosa, keratinized gingiva, hard palate, tongue dorsum, palatine tonsils, throat, saliva, supra- and sub-gingival plaques [75, 76]. The site with the most abundant *H. pylori* is hard palate, with 0.021% of average percent abundance, followed by buccal mucosa (0.004%) and throat (0.003%) [75]. The composition of oral microbiome is complex, niche-dependent, and distinct in health and disease [6]. Dental plaque and saliva are the most commonly used niches for oral sample collection (Tables 1 and 2). Inside the oral cavity, microorganisms are organized into different habitats that include those found on the keratinized gums, hard palate, and buccal mucosa (Group 1), those found on the tongue, tonsils, throat (posterior wall of the oropharynx) and in saliva (Group 2) and microorganisms in sub-gingival and supragingival plaque (Group 3) [94]. Microorganisms in the oral cavity are organized into different ecological niches; therefore, for the detection of *H. pylori* from the oral cavity, it would be ideal to collect samples from more than one niche.

**Table 2:** PCR detection of *H. pylori* in oral cavity: Diversity in amplified genes, study populations, and sample collection

References	Samples	Individuals	*Control Group*	Amplified gene											Types of PCR
				*16S rRNA*	*23S rRNA*	*ureA*	*ureAB*	*ureC*	*vacA*	*cagA*	*glmM*	*dupA*	*hsp60*	860-bp fragment	
Song et al. [27]	saliva, dental plaque, stomach	42 (adults)	No CG	–	–	–	–	–	–	–	–	–	–	X	Nested PCR
Mishra et al. [87]	Saliva, stool samples	137 (children—8 m—16 y) 108 (adults 17–60 y)	No CG	–	–	–	–	–	–	–	–	–	X	–	Nested PCR
Silva et al. [92]	Gastric biopsies saliva, dental plaque	30 (adults)	32 (adults)	X	–	–		–	–	X	–	–	–	–	Nested PCR
Eskandari et al. [88]	Gastric biopsies and dental plaque	67 (adults)	–	X	–	–	–	–	–	–	–	–	–	–	PCR
Fernandez-Tilapa et al. [33]	Saliva, dental plaque	200 (adults)	No CG	X	–	–	–	–	X	–	–	–	–	–	Semi-nested Nested PCR
Momtaz et al. [17]	Saliva, dental plaque, gastric biopsy, stool	300 (adults)	No CG	–	–	X	–	–	X	X	–	–	–	–	PCR
Rasmussen et al. [14]	Saliva, dental plaque, gastric biopsy	62 (adults)	No CG	–	–	–	–	–	X	X	–	–	–	–	PCR and Southern blotting
Román-Román et al. [29]	Saliva, gastric biopsy	196 (adults)	No CG	–	–	–	–	–	X	–	–	–	–	–	Conventional Nested PCR
Amiri et al. [65]	Dental plaque	45 (adults)	No CG	–	–	–	–	X	–	–	–	–	–	–	PCR and LAMP
Ismail et al. [80]	Dental plaque, gastric biopsy	49 (adults)	Same group after endoscopic examination	–	–	–	–	–	–	–	–	–	–	X	Single-step PCR Nested PCR
Bicak et al. [28]	Saliva, dental plaque	70 (children 5–16 y )	30 (children)	X	X	–	–	–	–	–	–	–	–	–	Real-time PCR
Castro-Muñoz et al. [83]	Saliva	162 (children 0–5 y )	No CG	X	–	–	–	–	–	–	X	–	–	–	PCR
Abu-lubad et al. [90]	Dental plaque	60 (adults)	No CG	X	–	–	–	–	–	–	–	–	–	–	PCR
Wongphutorn et al. [31]	Saliva, stool samples	110 (adults)	No CG	X	–	X	–	–	–	–	–	–	–	–	Semi-nested PCR, real time PCR
Nomura et al. [43]	Inflamed pulp specimens	131(adults)	No CG	–	–	X	–	–	–	–	–	–	–	–	Nested PCR
Iwai et al. [25]	Dental pulp, Dental plaque, Saliva, Urine	192 (adults)	No CG	–	–	X	–	–	–	–	–	–	–	–	Nested PCR
Kadota et al. [11]	Dental Pulp, Dental plaque of extracted tooth, saliva	39 (adults)	No CG	–	–	X	–	–	–	–	–	–	–	–	Nested PCR
Mallikaarachchi et al. [81]	Dental plaque	38 (adults)	33 (adults)	X	–	X	–	–	–	–	–	–	–	–	PCR
Nagata et al. [26]	Saliva, dental plaque, tongue coating	88 (adults)	No CG	–	–	–	–	–	–	–	–	–	–	X	Single-step PCR Nested PCR
Jara et al. [49]	Oral mucosa cheek, saliva	41 (adults)	21 (age 19–26)	–	–	–	–	–	–	X	–	–	–	–	Conventional PCR Nested PCR
Mehdipour et al. [91]	Dental plaque	36 (children 6–12 y)	36 (children 6–12 y)	X	–	–	–	–	X	X	–	X	–	–	Conventional PCR
Wongsuwanlerte et al. [16]	Saliva, Dental plaque, gastric biopsy	41 (adults)	No CG	–	–	–	–	–	X	X	–		–	–	Conventional Real-time PCR
Moosavian et al. [71]	Dental plaque, gastric biopsy	106 (adults)	No CG	X	–	–	X	–	X	X	–		–	–	Uniplex PCR

*CG:* control group, *PCR:* polymerase chain reaction, *y*: years

Microorganisms in the oral cavity may disrupt gastric homeostasis leading to inflammation and carcinogenesis [36]. Research suggests that the inflammation caused by bacteria in the gums and bones can harm the entire body or worsen other systemic diseases [37, 38]. Although the oral cavity is the primary route for *H. pylori* to enter the human gut, it is not yet clear how these bacteria adapt to the oral environment [39]. *H. pylori* is a microaerophilic bacterium that requires a high level of CO_2_ for optimal growth and survival [40–42]. *H. pylori* requires O_2_ for growth, and it is highly sensitive to atmospheric O_2_ levels. Functional gene groups exhibit differential regulation by O_2_ tension [42], and inflammasome activation by *H. pylori* is enhanced under low oxygen conditions [40]. *H. pylori* can survive within microaerophilic environments such as dental plaque biofilm in caries and periodontal pockets, and it can adhere to human dental pulp fibroblast cells [43] Synergistic interaction with oral microorganisms and transition to a viable but non-culturable (VBNC) or dormant state may also help *H. pylori* adapt to adverse conditions in the oral cavity [44]. The interaction between *H. pylori* bacteria and Candida yeast can be an example of a symbiotic relationship between the two. A study found fragments of *H. pylori* genes, vacA s1s2, and ureAB, in the total DNA of oral yeasts [45]. The intracellular presence of *H. pylori* in oral yeasts suggests that Candida yeast may contribute to the re-inoculation of *H. pylori* bacteria in the stomach or transmission to a new host [46].

Despite the separation between the oral and gut environments, it has been shown that more than half of the microbial species commonly found in both sites translocate from the oral cavity to the gut, even in healthy individuals [7]. Gastric *H. pylori* infection disturbs the oral microbiome [47], and the interactions between *H. pylori* and the oral microbiome may act through co-aggregation, endosymbiosis, and the formation of a symbiotic biofilm. Gastric eradicating *H. pylori* can also affect the oral microbiota [9]. The β diversity and composition of oral microbiota varied significantly among patients who had successful and failed gastric *H. pylori* eradication treatment. This suggests that changes in oral microbiota may play a role in the therapeutic effects of antibiotic therapy targeting *H. pylori* [48]. Detection of oral *H. pylori* could help monitor patients with no gastrointestinal symptoms of *H. pylori* earlier than the invasive approaches and could complement the invasive diagnosis and follow-up of patients [49]. Periodontal therapy can have a positive impact on the treatment of gastric *H. pylori*. Patients who received both periodontal treatments had a higher eradication rate, particularly those who had both oral and gastric *H. pylori*. Additionally, patients who underwent periodontal therapy had a higher non-recurrence rate compared to those who did not receive basic oral therapy, and these benefits were observed even after long-term follow-up [50].

Gastroesophageal reflux, poor oral hygiene, and frequent vomiting are conditions that can facilitate oral colonization, and the environment and lifestyle can be decisive for *H. pylori* colonization [33]. Poor oral hygiene is a factor considered in the recurrence of *H. pylori* infection, as the biofilm provides an ideal pH, temperature, and microaerophilic environment necessary for its survival [28]. The coccoid form may be a quiescent state of *H. pylori* in the oral cavity, but stressful situations, such as exposure to antibiotics and gastric acid, may cause oral coccoid *H. pylori* to transform into spiral-shaped bacteria [48]. Bacteria interact with each other in oral biofilms to survive in the oral environment. The aggregation of multiple bacterial species helps them colonize the oral cavity. Likewise, *H. pylori* may use the biofilm formed by *Streptococcus mutans* to survive and colonize the oral cavity. Therefore, preventing *Streptococcus mutans* infection in childhood and establishing habits such as good oral hygiene and sucrose restriction can be effective in preventing *H. pylori* infection [51]. A study investigated the effect of *H. pylori* culture supernatant on *S. mutans* and *Streptococcus sanguinis* dual-species biofilm and demonstrated that the inhibition rate evaluated through colony-forming units (CFU) exerted on *S. sanguinis by H. pylori* supernatant was statistically significantly higher than that exerted on *S. mutans*. However, in a dual-species biofilm model, *S. mutans* showed a superior competitive advantage over *S. sanguinis* under *H. pylori* supernatant treatment. The results from gene expression assays indicated that *H. pylori* supernatant can stimulate the production of mutacin and increase the acidogenicity of *S. mutans*, creating an environment that favors the growth of *S. mutans*, which becomes the dominant bacteria [52].

The digestive system, including the oral cavity, has a layer of mucus with glycoproteins called mucins as an important component. Mucins and other salivary components play a crucial role in acquired pellicle formation, and MUC1, MUC4, MUC19, MUC5B, and MUC7 are found in the oral cavity, with the last two being the most important dominants. Adhesins are proteins that mediate the adhesion of *H. pylori*. The first essential adhesin for *H. pylori* is BabA (blood group antigen-binding adhesin A) has a strong affinity with MUC5AC, mucin secreted in the stomach environment, and with MUC5B, proline-rich proteins and salivary agglutinins (gp-340). The second essential adhesin is SabA (sialic acid-binding adhesin), which in more neutral pH conditions, such as in the oral cavity, plays a fundamental role in the colonization of the oral mucosa by *H. pylori* [35, 53]. NapA (neutrophil-activating protein) is the third adhesin expressed by almost all strains of *H. pylori* and can bind sulfur oligosaccharides in saliva [53]. By maintaining a low abundance of specific oral bacteria (e.g., *Fusobacterium nucleatum, Porphyromonas gingivalis*, and *Tannerella forsythia*), immediate therapy in the periodontium and its related diseases may decrease the adhesion of oral *H. pylori* and improve the oral environment, leading to a reduction in the recurrence of gastric infections caused by *H. pylori* [48]. The structure of the salivary microbiota community differs among individuals infected and not infected with *H. pylori*. However, the eradication therapy does not alter the abundance, but the bacterial composition of the salivary microbiota [54]. Supragingival plaque consists mainly of early colonizers such as *Streptococcus* spp. and *Actinomyces* spp., which can modulate the physiology of oral *H. pylori.* The presence of *Streptococcus* spp., *Actinomyces* spp., and *Lactobacillus* spp. is related to the formation of microaerophilic conditions due to the intense saccharolytic metabolism associated with the fermentation of carbohydrates into organic acids, and subsequent acidification of the local environment and the maturation process of the supragingival plaque. It has been observed that *H. pylori* has a strong ability to coaggregate with *Fusobacterium* spp. naturally isolated from dental plaque (*F. nucleatum* and *F. periodontium*) [53]. The periodontopathic bacterial species were classified according to decreasing pathogenicity into three groups: the red complex (*P. gingivalis, Treponema denticola, and Tannerella forsythia*), the orange complex (*Prevotella intermedia, Prevotella nigrescens*, and* Campylobacter rectus*), and the green complex (*Capnocytophaga ochracea, Capnocytophaga sputigena, Aggregatibacter actinomycetemcomitans*, and *Eikenella corrodens*). A study demonstrated that the number of red complex species was significantly higher in *H. pylori*-positive individuals than in negative individuals. However, the number of orange and green complex species was significantly lower in positive individuals than in *H. pylori*-negative individuals [11].

The physical environment of the oral cavity differs considerably from that of the stomach because of the direct connection to the outside environment; chemically, a lower CO_2_ concentration and a higher O_2_ concentration are detected in the oral cavity than in the stomach. The microbial composition, unstable oral temperature, and mechanical scouring within the oral cavity also contribute to the differences between the two niches, mouth and stomach [44]. The presence of *H. pylori* in dental plaque is more frequent in molars compared to premolars or incisors. This may be due to the fact that the amount of oxygen exposure decreases gradually from incisors to molars, which creates an environment conducive to the growth of *H. pylori* in the molar region [55]. Eliminating *H. pylori* from the mouth through effective oral hygiene can increase the success rate of eradicating *H. pylori* and prevent its recurrence, which may be considered an additional treatment option for gastric *H. pylori* eradication therapy [35].

The microorganisms present in the oral cavity can influence the microbiological balance of the stomach, and reciprocally, the stomach microbiota can affect the microbial homeostasis of the oral cavity; therefore, it is important to better understand the oral bacterial community and its dynamic relationship with *H. pylori* when it is present in the oral cavity. Analyzing the frequency of *H. pylori* orally is not only important if there is a relationship between colonization in two locations (mouth and stomach) since *H. pylori* in the oral cavity can cause an imbalance in the microbiome, as well as remodel the oral ecosystem.

## Methods to identify oral *H. pylori*

**Fig. 1:**
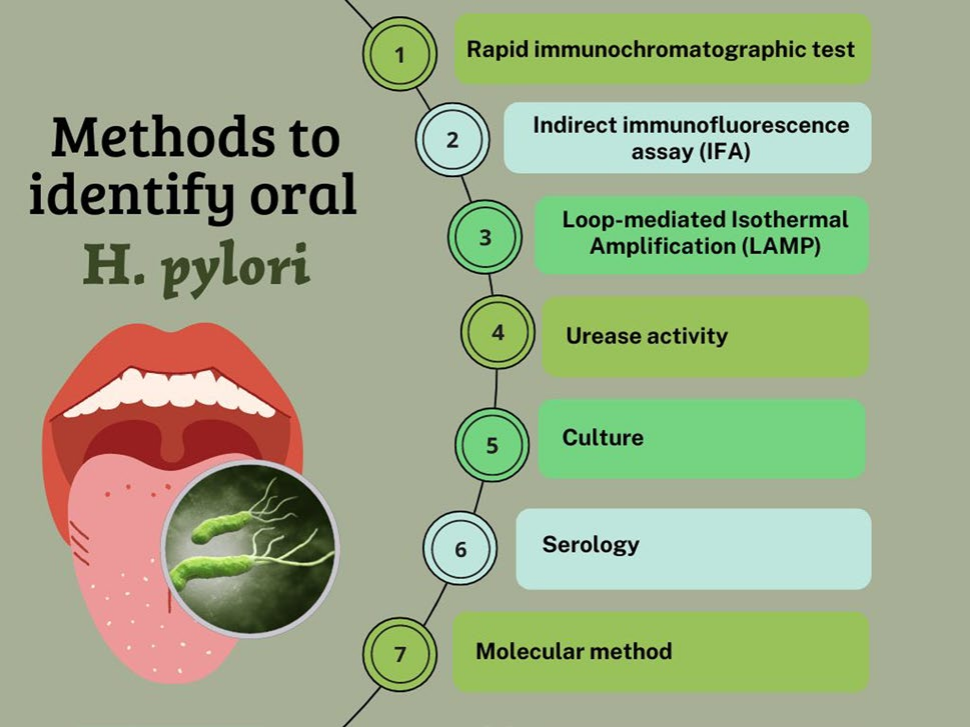
Overview of noninvasive methods for *H. pylori* identification in oral samples

There are two types of diagnostic methods to identify gastric *H. pylori* infections: invasive and noninvasive approaches. The invasive approach includes (endoscopy, histology, culture, and molecular methods) and noninvasive (urea breath test (UBT), stool antigen test (SAT), antibody detection, and molecular approaches) [56, 57]. Samples are collected in the oral cavity using noninvasive methods, and there are various techniques to detect the presence of *H. pylori* in the oral cavity (Fig. 1). There is a growing demand for noninvasive diagnostic methods to avoid the discomfort caused by the endoscopic examination required for sample collection [58]. The detection of *H. pylori* in a noninvasive sample, such as saliva or oral mucosa, without performing a complex procedure such as endoscopy, could be implemented as an excellent complementary diagnostic tool [49]. The fastidious nature of *H. pylori* makes its isolation challenging; thus, prevalence estimates might vary based on distinct methods and sample types [31]. Due to variations in the methods employed to detect *H. pylori*, differences in the studied population, oral hygiene status, tests with differing sensitivity and specificity, and variations in the type and amount of samples collected, it is difficult to compare the detection rate and quantity of *H. pylori* in dental biofilm and saliva across different studies [28].

### Rapid immunochromatographic test

*H. pylori* antigens can be identified in saliva by immunochromatographic assays [59]. Rapid immunochromatographic antigen tests of saliva (HPS) employed monoclonal antibodies that were used to identify oral *H. pylori*, as well as compare with urea breath test (UBT) C^13^. The positive rate of oral *H. pylori* among all groups was 51.96%. The prevalence of oral infection by *H. pylori*, concerning age (< 45–89 years), was higher in younger groups and lower in the elderly and is associated with the number of teeth [60]. Patients had been diagnosed using the saliva *H. pylori* antigen test (HPS), the *H. pylori* flagellin test (HPF), the UBT C^13^, and the polymerase chain reaction (PCR) test. These tests were subsequently confirmed through saliva culture. Both antigen tests were strong indicators of the presence of *H. pylori* antigen in the mouth. The tests, with monoclonal antibodies, recognized two *H. pylori* antigens (urease and flagellin) in symptomatic and asymptomatic people. The presence of *H. pylori* in saliva has been observed even when patients test negative for UBT C^13^. Thus, in the absence of a stomach infection, patients may still exhibit the *H. pylori* antigen in the mouth [59]. Researchers used saliva samples to detect antigens of* H. pylori* using the noninvasive “one-step *H. pylori* saliva antigen” (HPS) test. However, the study revealed a low detection rate of *H. pylori* urease antigen in saliva, with only 20% of the samples testing positive for *H. pylori* compared to 52.2% with the reference examination in gastric mucosa [61].

### Indirect immunofluorescence assay (IFA)

In a study with healthy asymptomatic individuals, the detection of *H. pylori* infection in saliva and stool samples was conducted using a combination of immunological and molecular techniques. The results showed positive *H. pylori* infection in 59.1%, 65.5%, and 51.8% of saliva samples, based on semi-nested PCR, SYBR green real-time PCR, and IFA, respectively. At least one of these methods detected positive results in 89.1% of saliva samples and 82.7% of stool samples [31]. In another study, the detection of *H. pylori* in asymptomatic individuals was done using saliva samples. Three methods were employed: nested PCR tests, SYBR green qPCR, and IFA. The results showed that the prevalence of *H. pylori* in saliva samples was 57% by nested PCR, 56% by SYBR green qPCR, and 50% by IFA test and the combination of these methods revealed that the nested PCR and qPCR tests are more sensitive in detecting *H. pylori* in saliva samples compared to the IFA test [62].

### Loop-mediated isothermal amplification (LAMP)

LAMP is a nucleic acid-based assay for identifying *H. pylori* in clinical specimens. It is quick, accurate, and cost-effective for diagnosing many infectious agents with good sensitivity and specificity [63]. The use of noninvasive sampling techniques like saliva, oral brushing, and fecal sampling for the detection of *H. pylori* using LAMP as point-of-care testing could be considered a rapid diagnostic test. The best results were observed using saliva samples (sensitivity 58.1%, specificity 84.2%, PPV 85.7%, NPV 55.2%, accuracy 68%), followed by oral brushing samples and fecal samples. The ability to detect 0.25 fg/μL of the *H. pylori* DNA genome from clinical samples shows its high sensitivity for pathogen diagnosis that the specificity and accuracy of LAMP are higher than conventional PCR and other immunological rapid tests. [64]. In another study, the prevalence of *H. pylori* was assessed in the dental plaques of patients with chronic periodontal diseases using LAMP and PCR. Results showed that the LAMP method was significantly more effective than PCR (with a chi-square P value < 0.05). Out of all the samples, one of the two tests was positive in 77.78% of cases, while neither LAMP nor PCR was positive in 22.22% of cases [65].

### Urease activity

Urease is an enzyme that hydrolyzes urea (carbamide) into ammonia and CO_2_ and is produced by several bacterial species including *H. pylori*. Urea is supplied in gingival crevicular fluid and salivary gland secretions in concentrations ranging from 3 to 10 mM in healthy individuals. Such concentrations can increase the pH (alkalization) of the dental biofilm and therefore can significantly neutralize the effects of glycolytic acidification on plaque [66]. Among the noninvasive techniques used to detect *H. pylori* in the oral cavity, the urease method is probably the most common choice, but the presence of other urease-producing bacteria in the oral flora may hinder the applicability of this method [67]. In one study tissue biopsy, saliva and oral swab samples were collected and tested for identification of *H. pylori* using urease test, culture media, and PCR [68]. Urease test was positive in 82.05% of saliva samples and 43.05% of oral swab samples. In another study, to determine the prevalence of periodontal disease and *H. pylori* colonization in dental plaque and the seroprevalence of *H. pylori* infection, a group of individuals were analyzed using a questionnaire, oral examination, dental plaque, rapid urease test (RUT), and serological examination for immunoglobulin G antibody for *H. pylori*. The results showed that 61.4% of the participants who had periodontal disease were RUT positive, while only 43.5% of the participants who did not have periodontal disease were RUT positive. The difference between the two groups was significant (*P* = 0.00007, OR = 2.07, 95% CI 1.42 < OR < 3.02), suggesting that periodontal disease increases the risk of *H. pylori* colonization within dental plaque [69]. In another different study, RUT was used on dental plaque samples to assess the presence of *H. pylori* and determine the role of oral *H. pylori* colonization and periodontal health in the development of recurrent aphthous stomatitis (RAS). The RUT positivity rate in the patient group was significantly higher than that in the control group, and the positivity was observed to be a significant risk factor for the development of RAS [70]. Analysis of the supragingival plaque in the later study, through RUT, in four different locations showed strong urease activity in the anterior region of the mandible. Teeth in the anterior mandibular region are characterized by a low rate of caries and a high prevalence of calculus related to low and high pH, respectively; therefore, the hydrolysis of urea may have a biological impact on periodontitis and caries [66]. Recently, it was found that there is a strong connection between the detection of *H. pylori* bacteria in the oral mucosa through RUT and PCR testing. The two tests showed optimal agreement in their results. However, there was no significant correlation between RUT and saliva PCR tests, and between oral mucosa PCR and saliva PCR results [49].

Urease activity was detected in coccoid forms of *H. pylori* both phenotypically using the rapid urease test and genotypically using the PCR method. The presence of urease activity showed that the transformation from the helical to the coccoid form influenced the urease activity independently of the transformation factor [5]. Using RUT in a microplate format as a method to test urease activity in oral bacterial strains in vitro, rapid and strong reactions were observed for *H. pylori* and *Campylobacter ureolyticus, Haemophilus parainfluenza*, and *Staphylococcus epidermidis* [66]. False-positive results of this test are possible in certain conditions because several organisms such as *Klebsiella pneumoniae, Staphylococcus aureus, Proteus mirabilis, Enterobacter cloacae, and Citrobacter freundii*, isolated from the oral cavity and/or stomach, also present urease activity as indicated in a different study [57].

### Culture

Many attempts to culture *H. pylori* from the oral cavity have failed due to its ability to exist in a non-cultivable coccoid form. As a result, some researchers believe that *H. pylori* may survive in the oral cavity in this form and can only be detected through non-culture methods [71]. However, a study investigated the colonization of  *H. pylori* in the presence of *S. mutans* using strains in the laboratory environment. According to the results, *H. pylori* tends to concentrate in the areas where *S. mutans* grows densely. This suggests that the location of the bacterium in the biofilm depends on the presence or absence of *S. mutans*. The number of *H. pylori* is significantly higher when both *S. mutans* and *H. pylori* are present compared to when *H. pylori* is alone in the cultures. In the former, there were 1.2 × 10^4^ colony-forming units (CFUs) of *H. pylori*, while in the latter, there were only 1.1 × 10^2^ CFUs of *H. pylori* (P < 0.001) [51].

### Serology

Serological tests are mainly based on the investigation of antibodies against *H. pylori.* Antibody classes including the immunoglobulins IgG, IgA, and IgM can also be measured in screening for *H. pylori* infection; however, IgG has shown more reliable results [56]. Tests for the detection of antibodies against *H. pylori* using urine and saliva samples have been reported in the scientific literature [72]. However, due to the lower concentration of antibodies in these samples compared to serum-based diagnostic methods, the investigation of antibodies against *H. pylori* is limited for the correct identification of the infection [56, 72]. Thus, serological tests can result in false negatives, as new infections can occur when antibody levels are not high enough. IgG antibodies appear approximately 21 days after *H. pylori* infection. After successful eradication treatment, IgG antibodies to *H. pylori* remained for several months. [72]. The main disadvantage of the serological approach is its inability to distinguish between current infection and previous exposure. Thus, an erroneous interpretation may occur, as IgG antibodies are still found for a few months after treatment; that is, a positive result may occur even after bacterial clearance treatment [57]. In another study, enzyme-linked immunosorbent assay (ELISA) was used to detect anti-*H. pylori* IgG and IgM in saliva and supragingival dental plaque samples and among the individuals tested**,** 62% of them were seropositive [33].

### Molecular methods

At the time of this writing, a total of 774 prokaryotic species of the human oral and nasal microbiome have been identified in the Human Oral Microbiome Database (HOMD), based on the 16S rRNA sequence phylogeny. Of them, 58% are officially named, 16% are unnamed but cultivated, and 26% are known only as uncultivated phylotypes [5, 73]. For *H. pylori,* DNA/RNA-based molecular techniques that have been used in diagnosing *H. pylori* infection include polymerase chain reaction (PCR), real-time PCR, droplet digital PCR (dd-PCR), fluorescent in situ hybridization (FISH), and next-generation sequencing (NGS) including 16S rRNA amplicon sequencing, metagenomics, and metatranscriptomic sequencings [77]. PCR can amplify specific regions of *H. pylori* in saliva, dental plaque, gastric biopsies, gastric juice, and stool. These regions include *vacA*, *cagA*, *ureA*, *glmM*, *hsp60*, 16S rRNA, 23S rRNA, and *ureC* (glmM) genes [56, 57]. Significant variations are observed in the populations studied, covering factors such as the absence of a control group in the study groups, oral health status, age diversity, varying sample sizes, as well as diversity in locations where the oral samples were collected. PCR is still the most used method for detecting *H. pylori* in the oral cavity. Table 2 summarizes the PCR-based studies surveyed in this review. It is difficult to compare the detection rate and quantity of *H. pylori* in oral samples due to differences in the PCR protocols (annealing temperature, number of cycles) used to detect *H. pylori*, as well as the primer sets used, size of the amplicon, and the genes chosen for amplification.

#### Polymerase chain reaction (PCR)

It is challenging to detect *H. pylori* with high accuracy in the oral cavity due to the presence of around 700 other oral bacterial species, despite the use of PCR assays [43, 78]. The oral cavity contains bacterial species closely related to *H. pylori*, such as *Campylobacter* and *Wolinella*, leading to false-positive results for *Helicobacter*. Thus, PCR methods for the detection of oral *H. pylori* should be interpreted with caution due to the presence of other microorganisms in the mouth that are phylogenetically related to *H. pylori* [79]. Sequencing PCR products can overcome this problem by identifying non-*Helicobacter* species that may have caused false positives [79]. PCR has a high sensitivity to detect DNA of a low abundant organism present in a clinical sample and can amplify target DNA from coccoid forms of *H. pylori* that are difficult to culture and identify histologically [80]. It is recommended to use a combination of two or more methods (i.e., nested PCR, qPCR) for the detection of *H. pylori* in saliva samples to avoid false-negative results. Discrepancies between the two PCR methods could arise due to two reasons: Firstly, the primers used in both methods were different, and secondly, the target gene sequences in the saliva samples may have variations compared to those present in the GenBank database. Therefore, it is advantageous to use different genes to confirm true-positive results for *H. pylori* detection [62]. The conflicting PCR results regarding the presence of *H. pylori* in the oral cavity may also be due to the different specificities and sensitivities of the primers used [80]. Oral biofilm samples from dental professionals and non-dental undergraduate students were evaluated, revealing a significant difference between the prevalence of *H. pylori* in the oral biofilms of dental undergraduate students with clinical exposure and non-dental undergraduate students without clinical exposure. In samples positive for the 16S rRNA gene of *H. pylori*, the prevalence was 28.95% in undergraduate dentistry students and 9.09% collected from non-dental undergraduate students. All samples were positive for the 16S gene. *H. pylori* rRNA in the PCR also tested positive for the urease gene [81]. Comparing the sensitivity of the nested PCR method with the single PCR method, it was found that the former had a higher sensitivity due to the use of two primer sets [43]. Another factor reported to underestimate the frequencies of *H. pylori* is the DNA extraction method used due to boiling and the low quality of the kit that has been used for extraction [65].

In one study, it was reported that after initial PCR amplification, no visible DNA band was observed in the dental plaque samples. However, after subjecting the PCR products to nested PCR, the results showed that some patients had *H. pylori* present in their dental plaque samples, suggesting that there was a low copy number of *H. pylori* DNA. Therefore, the use of single-stage PCR is not reliable for detecting *H. pylori* in oral samples and nested PCR would be a more efficient method for this purpose [80]. In another study that uses nested PCR utilized to investigate the presence of *H. pylori* in dental plaque and saliva, the results showed that the bacteria were identified in dental plaque samples at a higher rate (97%) compared to saliva samples (55%). Furthermore, in this sample study, the prevalence of *H. pylori* in dental plaque was found to vary depending on the region of the oral cavity from where the sample was collected [55]. The variation in detection rates in dental plaque and saliva samples makes it difficult to recommend PCR as the gold standard method [67].

Among the three niches (saliva, teeth, and tongue), the supragingival biofilm adhered to teeth was the most common site for detecting *H. pylori*. The lower incisor was the primary site for detecting *H. pylori* in supragingival biofilms followed by the upper incisors, lower left molars, and upper right molars. The percentage of samples that tested positive for *H. pylori* increased when nested PCR was used [26]. The supra- and sub-gingival plaque samples demonstrated higher levels of *H. pylori* than the saliva and tissue gastric biopsy samples [16]. When analyzing samples that contain both saliva and stool combined, the use of semi-nested PCR targeting *vacA* and real-time PCR targeting the 16S rRNA gene enhanced the detection rate. Relying on a single target alone may occasionally result in failure to amplify [31].

A new PCR method was developed to investigate the distribution of *H. pylori* in saliva and inflamed pulp. The method used highly conserved sequences from the complete genomes of 48 *H. pylori* strains to design five sets of primers. The primer set ureA-aF/ureA-aR was the most sensitive and was used for all further analyses, and then, the detection of *H. pylori* in oral specimens was done by analyzing clinical specimens using the primer set ureA-aF/ureA-aR [82]. In a study conducted on asymptomatic children, samples taken from the cheek region were tested using PCR for the 16S rRNA and *glmM* genes. It was found that out of 162 samples, 21 were positive for *H. pylori* and that the prevalence of *H. pylori* infection increased with age [83]. A study compared *H. pylori cagA* and the *vacA* allelic status among strains isolated from saliva, dental plaque, gastric biopsies, and stool samples in the same patient with dyspepsia manifestations [17]. Of the saliva samples positive for *H. pylori*, all were *cagA* positive, and there was no association between *H. pylori* genotypes in saliva and clinical outcomes. All patients with positive *H. pylori* in their saliva had a positive PCR for gastric biopsy samples simultaneously.

The detection rate of *H. pylori* varied with each primer set used. The frequency of *H. pylori*-positive samples was 70.5% (43/61) using nested PCR (16S rRNA), but only 9.8% (6/61) using single-step PCR (860 bp) and none of the samples tested positive for the urease A gene. However, in this study it was not possible to establish a statistically significant association neither with the presence of *H. pylori* and periodontitis status nor with gender [67]. In the study, using RT-PCR, dyspeptic children (aged 5–16) had higher levels of the 16S rRNA and 23S rRNA genes in their dental biofilm and saliva samples compared to the control group. The detection of *H. pylori* in the dental biofilm of the gastric *H. pylori*-positive group was significantly higher than that of the negative and control groups. The detection rate of this microorganism in dental biofilm and saliva samples, with the amplification of both 16S rRNA and 23S rRNA genes, showed that the detection rate increased when using only one gene amplification but decreased when both genes were amplified for bacterial identification [28].

#### Next-generation sequencing (NGS)

NGS for the bacterial 16S rRNA gene has high sensitivity and specificity (95–100%) and presents quantitative microbiome data showing interspecies interaction, and prediction of antibiotic resistance. However, this method is expensive and requires skills, and false positives occur due to the cutoff value and lack of negative controls, in addition to being unable to distinguish between live and dead bacteria, and present difficulty in separating *H. pylori* from closely related species [77]. To establish the bacterial composition, abundance, and structure of the salivary microbiome in people with and without active *H. pylori* infections, performed 16S rRNA gene amplicon sequencing using V3-V4 demonstrated that both *H. pylori* infection and *H. pylori* therapy eradication caused changes in the community and structure of the oral microbiota. The abundance of salivary microbiota measured by the number of OTUs collected was similar in uninfected and infected individuals. Bacterial diversity in saliva is similar between *H. pylori*-uninfected people and *H. pylori*-infected people, but salivary microbiota community structures were different [54].

#### Multilocus sequence typing (MLST)

MLST is a method of identifying bacterial isolates by sequencing seven housekeeping genes [84, 85]. To analyze and compare the differences in genotype and explore the genetic relationship between *H. pylori* in the stomach and mouth of patients with *H. pylori* infection, the technique MLST can be utilized. The results indicate a large sequence of diversity between strains of oral and gastric origin in most samples analyzed suggesting that the oral and gastric *H. pylori* probably had completely different origins [34].

#### High-throughput multiplex genetic detection system (HMGS)

HMGS assay is a high-throughput technique that quickly identifies and quantifies *H. pylori* while also analyzing virulence and drug resistance. It can also distinguish mixed infections with different resistant genotype strains [86]. Noninvasive HMGS exhibited high levels of accuracy for the identification of *H. pylori* in oral specimens (saliva, mouthwash, and dental plaque) when compared to conventional methods (urease e qPCR). Relative quantitative analysis of *H. pylori* infection in oral samples showed that the detection peak area of noninvasive HMGS increased with the increase in UreC concentration. Consistent with *H. pylori* loads in oral samples, it was reported that positive detection rates of *H. pylori* virulence genotypes in saliva were higher than those in mouthwash and dental plaque [24]. According to these authors, the positive detection rates of cagA, iceA1, luxS, and oipA in saliva were significantly higher than those in mouthwash and dental plaque (p < 0.05). Furthermore, the positive detection rates of vacA s1m2, cagA, iceA2, and oipA in mouthwashes were significantly higher than those in dental plaque [24].

## Conclusions

Understanding if the oral cavity is a reservoir for *H. pylori* is important for clarifying the transmission and reservoir dynamics of *H. pylori*. Establishing accurate noninvasive methods, such as those using samples collected from the mouth, could lead to the development of a complementary diagnostic tool for *H. pylori*. This is particularly crucial if accurate diagnostic methods are developed and a correlation between the presence of *H. pylori* in the oral cavity and gastric mucosa is established. For detecting *H. pylori* in the oral cavity, PCR is still the most used method and it is still the most easily accessible. However, due to the diversity of amplified genes and variations in the protocols used in the reactions, standardizing the *H. pylori* detection protocol is challenging. Eradicating *H. pylori* from the oral cavity can help prevent transmission and re-colonization of the stomach, provided that the strains are identical at both sites and that effective treatment strategies are employed for both locations. Continued studies of *H pylori* in the oral cavity, including its role in the oral microbiome, prevalence, abundance, and genomic similarity to gastric strains, will deepen our understanding of this species. This may eventually provide new clinical insights and improve the treatment of *H. pylori* infection, leading to a shift in the understanding of oral and gastric diseases.

## Data Availability

No datasets were generated or analyzed during the current study.
